# Evaluation of Citrated Plasma After Thawing for Routine Coagulation Testing

**DOI:** 10.7759/cureus.40023

**Published:** 2023-06-06

**Authors:** Sathwik Reddy, Tushar Sehgal, Gunvanti Rathod, Shailaja Prabhala, Prafull Kamble, Sudhanshu Shekhar, Parag Patil

**Affiliations:** 1 Pathology and Laboratory Medicine, All India Institute of Medical Sciences, Bibinagar, IND; 2 Laboratory Medicine, All India Institute of Medical Sciences, New Delhi, IND; 3 Physiology, All India Institute of Medical Sciences, Bibinagar, IND; 4 Pathology and Laboratory Medicine, All India Institute of Medical Sciences, Kalyani, IND; 5 Pathology and Laboratory Medicine, All India Institute of Medical Sciences, Binibagar, IND

**Keywords:** international normalized ratio (inr), activated partial thromboplastin time (aptt), prothrombin time (pt), thawing, citrated plasma, coagulation testing

## Abstract

Objective: We aim to find the time in which a thawed citrate plasma sample that was preserved can be analyzed for routine coagulation testing without losing precision.

Methods: Whole blood samples from 30 healthy volunteers were collected in 3.2% sodium citrate vacutainer and centrifuged to separate platelet-poor plasma. Each sample was then aliquoted, one aliquot was used immediately for prothrombin time (PT)-international normalized ratio (INR) and activated partial thromboplastin time (APTT), four were stored at -20°C, and four were stored at -80°C for 24 hours. After 24 hours, the aliquots were taken out and thawed at 37°C in water bath and analyzed after 15, 30, 60, and 120 minutes.

Statistical analysis: Data were presented as mean with standard deviation (SD). Repeated measures ANOVA with Tukey post-hoc test was performed for multiple comparisons. All analysis was done using GraphPAD Prism 8.0 software (GraphPad Software, San Diego, California, USA).

Results: In the case of PT and INR, no statistically significant difference was found between the mean values after thawing for 120 minutes when compared with the mean baseline value. However, the APTT showed a statistically significant difference (p = 0.0232) after 30 minutes of thawing when the sample was stored at -20°C. Furthermore, a statistically significance difference (p = 0.0001) was found after 60 minutes of thawing when the samples were stored at -80°C.

Conclusion: Plasma samples for the PT and INR may be accepted for assessment up to 120 minutes, when stored at -20°C and -80°C for 24 hours. In the case of APTT, the plasma sample can be used for assessment up to 30 minutes after thawing when stored at -20°C and up to 60 minutes when stored at -80°C.

## Introduction

The coagulation cascade involved in the hemostatic process is the mechanism of conversion of plasma fibrinogen to the solid mass of fibrin [1]. It helps in limiting blood loss from an injured vessel. The intrinsic and extrinsic pathways of the coagulation cascade based, both leading to a common pathway, activated factor X in the presence of calcium to form a stable clot. Prothrombin time (PT) and activated partial thromboplastin time (APTT) tests are two tests that are routinely used to diagnose and quantify disorders associated with the two coagulation pathways [2]. PT is the time taken in seconds by citrated plasma to clot after the addition of tissue thromboplastin and calcium and is a measure of the extrinsic and common pathway. It is used to monitor warfarin therapy and evaluate liver function. Due to variations in the sensitivity of thromboplastin reagents, PT is reported as the international normalized ratio (INR). APTT is the time taken in seconds for the citrated plasma to clot in the presence of a surface activator and calcium. It is a measure of the intrinsic and common coagulation pathway. It is used as an important test in the diagnosis of hemophilia A and B, detection of coagulation inhibitors, and monitoring of heparin therapy. The PT, APTT, and INR tests form a panel of tests that are ordered most extensively as a part of coagulation studies and are the most frequently performed tests in laboratories to help clinicians in patient care decisions [3].

PT, INR, APTT, factor X, and lupus anticoagulant testing are affected by freezing and thawing conditions [4]. Earlier, storage of blood specimens for coagulation test was not recommended for longer time, and tests were advised to be performed within 24 hours of the collection, with the samples stored at 4°C. In the last few years, several studies have focused on the transport and storage of plasma samples for PT, APTT, and INR [5]. They have reported that PT, APTT, dilute Russell viper venom time (DRVVT), activated protein C resistance (APCR), and D-dimer can be stored for two weeks at -20°C without compromising clinical interpretation in both healthy individuals and individuals with coagulopathy. These recent advancements have given time for small laboratories to facilitate sample processing from off-site clinics. Some recent studies also suggested that samples for PT and INR could be safely stored for ≤24 hours both at 4°C and 25°C and APTT for ≤12 hours at 4°C and ≤8 hours at 25°C [6]. The patient plasma samples for PT, INR, and APTT tests could be safely stored for up to 36 hours in the freezer. Another study suggested that the samples for PT and INR tests could be safely stored for up to 24 hours while the samples for APTT deteriorated at 12 hours when stored in the refrigerator, whereas all patient samples for PT, INR, and APTT tests deteriorated at 12 hours at room temperature [7]. As a recommendation, plasma samples should be analyzed up to 12 and 24 hours when stored at 4°C and 25°C, respectively, and up to two weeks when stored appropriately at -20°C.

Storing samples at -20°C and -80°C for a limited time period has been substantiated; however, there are many aspects that one should keep in mind when storing samples at lower temperatures. It is very important to understand the stability of a patient’s plasma along with its coagulation proteins to maintain the reliability and accuracy of the laboratory results of coagulation assays. One of the most important pre-analysis parameters is the stability of the sample after thawing and the acceptable transition time from -20°C or -80°C to 37°C before performing the test. Studies seldom aim to account for the effect of thawing on the analysis of frozen citrated plasma samples and to ascertain the safe time within which the plasma can be studied reliably. One crucial factor to be noted here is that the automated coagulation analyzers are usually batch analyzers, where the first sample in line gets analyzed immediately while it takes a considerable time for the samples at the end to be in the analysis chamber. This delay could also account for errors when the samples are to be run for coagulation studies after thawing.

In this study, we have tried to explore the stability of stored frozen citrated plasma, stored for 24 hours at -20°C and -80°C, after thawing for the analysis of PT, INR, and APTT, and to evaluate an acceptable transition time up to which the samples can be analyzed for accurate and precise results.

## Materials and methods

The study was performed under the guidance of the Department of Pathology and Laboratory Medicine at the All India Institute of Medical Sciences (AIIMS), Bibinagar, Hyderabad, India. Ethical clearance was obtained from the Institutional Ethics Committee of AIIMS Bibinagar (approval number: AIIMS/BBN/IEC/JULY/2022/180). A total of 30 healthy volunteers, more than 18 years of age, were recruited into the study, and whole blood samples were obtained from them using the standard aseptic phlebotomy procedure. The blood samples were collected in blue top blood collection tubes containing 3.2% sodium citrate anti-coagulant in a ratio of one part of anti-coagulant to nine parts of whole blood. Volunteers with any history of coagulation disorders and/or on any medications affecting blood coagulation, such as heparin, warfarin, and aspirin, were excluded from the study. Hemolyzed, lipemic, and icteric samples were also excluded.

The samples were then centrifuged at 2000 g for 10 minutes to obtain platelet-poor plasma (PPP). PT, INR, and APTT tests were performed on the PPP on ECL 760 Fully Automated Coagulation Analyzer (Erba Mannheim, manufactured at Erba Lachema s.r.o., Brno, Czech Republic). PT and INR were measured using the reagents Erba Protime LS® (Erba Mannheim, manufactured at Erba Lachema s.r.o.), whereas APTT was performed using reagents Erba Actime® and Erba Calcium Chloride® (Erba Mannheim, manufactured at Erba Lachema s.r.o.). Two control levels, normal and abnormal, were run before starting the analysis. The reference limits for the analysis, as specified by the manufacturer, were 11.5-14.6 seconds for PT and 0.93-1.16 seconds for INR with an international sensitivity index (ISI) of 0.98. The reference interval for APTT was 21.7-29.3 seconds.

The separated PPP from each sample was aliquoted into nine microcentrifuge tubes, and one aliquot of PPP was used immediately for the PT, INR, and APTT tests. The obtained results were taken as baseline values (zero hours). Four aliquots were stored at -20°C, and four were stored at -80°C for 24 hours. After 24 hours, the aliquots were taken out and thawed at 37°C in a water bath and analyzed after 15, 30, 60, and 120 minutes. The results were recorded corresponding to the storage temperature and time at which the samples were analyzed.

Statistical analysis

The data were reported based on its qualitative or quantitative nature. Qualitative data were analyzed as percentages and frequencies, whereas quantitative data were presented as the mean with standard deviation (SD). The repeated measures ANOVA was used to calculate any statistical difference between the groups. A Tukey post-hoc test was performed for the multiple comparisons. A p-value of less than 0.05 was considered significant. All analyses were done using GraphPAD Prism 8.0 software (GraphPad Software, San Diego, California, USA).

## Results

**Figure 1:**
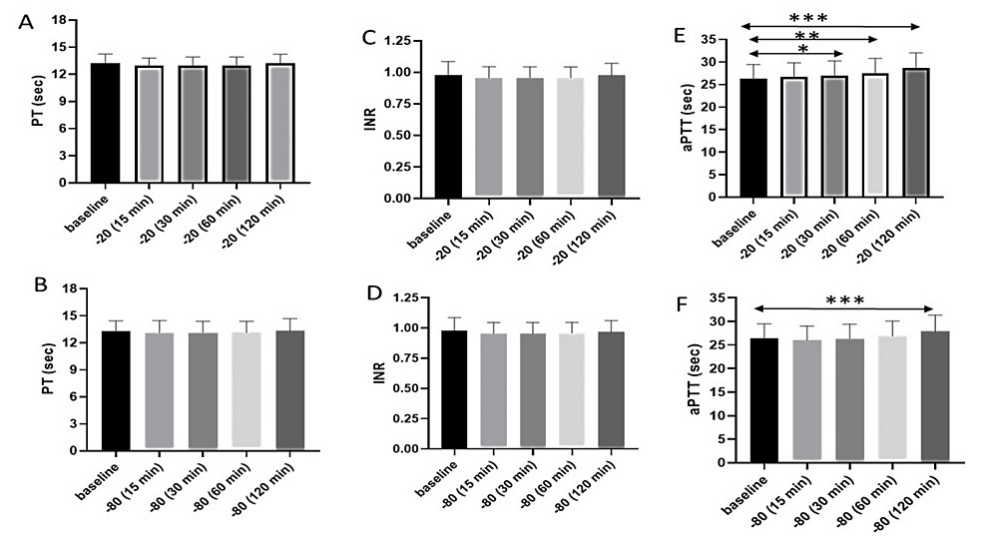
Representation of the PT-INR and APTT (A) Bar diagram represents the PT value at the baseline (without any storage), 15 minutes, 30 minutes, 60 minutes, and 120 minutes after thawing when the samples were stored at -20℃ for 24 hours. (B) Bar diagram represents the PT value at different time points when the samples were stored at -80℃ for 24 hours. (C) INR value at different time points when the samples were stored at -20℃ for 24 hours. (D) INR value at different time points when samples were stored at -80℃. (E) APTT value at different time points when the samples were stored at -20℃ for 24 hours. (F) APTT value at different time points when the samples were stored at -80℃ for 24 hours. Asterisks indicate the level of statistical significance: * p ≤ 0.05, ** p ≤ 0.01, *** p ≤ 0.001. PT: prothrombin time; APTT: activated partial thromboplastin time; INR: international normalized ratio

Samples from 30 healthy volunteers were analyzed. The male-to-female ratio was 5:1, with a mean age of 21 years (range: 18-27 years). The mean of the baseline values of PT was 13.37 seconds. The mean value of the samples stored at -20°C and analyzed after thawing for 15 minutes was 13.15 seconds. This difference was statistically not significant from the mean of the baseline value. Similarly, the mean value of samples stored at -20°C and analyzed after thawing for 30 minutes was 13.15 seconds, that of samples analyzed after thawing for 60 minutes was 13.18 seconds, and that of samples analyzed after thawing for 120 minutes was 13.43 seconds (Figure 1A). In all the cases, the difference was statistically not significant from the mean of the baseline values. Likewise, the mean value of the samples stored at -80°C and analyzed after thawing for 15, 30, 60, and 120 minutes was 13.11, 13.12, 13.14, and 13.33 seconds, respectively. However, no statistically significant changes were found between the values when compared with the baseline value (Figure 1B).

The mean of the baseline value of the INR was 0.98. The mean INR value of the samples stored at -20°C and analyzed after thawing for 15, 30, 60, and 120 minutes was 0.96, 0.96, 0.96, and 0.98, respectively, none of which were statistically different from the baseline value (Figure 1C). Similarly, the mean INR value of the samples stored at -80°C and thawed was 0.95 after 15 minutes, 0.95 after 30 minutes, 0.96 after 60 minutes, and 0.97 after 120 minutes. No statistically significant difference was found between the values when compared with the mean of the baseline values (Figure 1D).

The mean of the baseline values of APTT was 26.39 seconds. The mean value of the samples stored at -20°C and analyzed after thawing for 15 minutes was 26.7 seconds. This difference was statistically not significant from the mean of the baseline values (p-value = 0.8694). Moreover, the mean value of the samples stored at -20°C and analyzed after thawing for 30 minutes was 27.03 seconds, which was not significantly different from the mean of the baseline values (p-value = 0.329). However, the mean values of the samples stored at -20°C and analyzed after thawing for 60 minutes (p-value <0.0232) and 120 minutes were 27.54 and 28.73 seconds, respectively, both of which were statistically different from the mean of the baseline values (p-value <0.0001) (Figure 1E). On the other hand, the mean value of the samples stored at -80°C and analyzed after thawing for 15, 30, and 60 minutes was 26.0, 26.34, and 26.89 seconds, respectively. These values were not statistically different when compared with the baseline APTT values. However, the mean value of the samples stored at -80°C and analyzed after thawing for 120 minutes was 27.86 seconds. This difference was statistically significant from the mean of the baseline values (p-value =0.0001) (Figure 1F). All the data on the mean and p-values are summarized in Table 1.

**Table 1:** Mean values of the PT-INR and APTT PT, APTT, and INR mean values at the baseline (no storage), 15 minutes, 30 minutes, 60 minutes, and 120 minutes since thaw at 37℃. All the samples were stored at -20℃ and -80℃ for 24 hours before performing the experiment. Data were analyzed using repeated measures ANOVA. PT: prothrombin time; APTT: activated partial thromboplastin time; INR: international normalized ratio; ANOVA: analysis of variance

PT; Mean of the baseline values: 13.27 seconds					
No.	Stored at (temperature)	Time since thaw	Mean	Difference from mean of baseline	P value
1	-20°C	15 minutes	13.15	0.1161	0.4259
2		30 minutes	13.15	0.1226	0.3037
3		60 minutes	13.18	0.08710	0.7396
4		120 minutes	13.43	0.1613	0.6320
5	-80°C	15 minutes	13.11	0.1548	0.4322
6		30 minutes	13.12	0.1516	0.3404
7		60 minutes	13.14	0.1290	0.6271
8		120 minutes	13.33	0.06452	0.9750
INR; Mean of baseline values: 0.9799					
1	-20°C	15 minutes	0.9557	0.02419	0.1149
2		30 minutes	0.9564	0.02352	0.0957
3		60 minutes	0.9558	0.02406	0.0943
4		120 minutes	0.9771	0.002774	0.9990
5	-80°C	15 minutes	0.9535	0.02639	0.0903
6		30 minutes	0.9536	0.02623	0.0943
7		60 minutes	0.9562	0.02371	0.0954
8		120 minutes	0.9672	0.01271	0.7694
APPT; Mean of baseline values: 26.39 seconds					
1	-20°C	15 minutes	26.70	0.3097	0.5190
2		30 minutes	27.03	0.6419	0.3294
3		60 minutes	27.54	1.152	0.0232
4		120 minutes	28.73	2.339	0.0001
5	-80°C	15 minutes	26.34	0.0535	0.2181
6		30 minutes	26.43	0.0548	0.9972
7		60 minutes	26.89	0.5000	0.0772
8		120 minutes	27.86	1.468	0.0001

## Discussion

The PT, APTT, and INR tests are parts of hematological studies and are the most frequently performed tests in laboratories. These tests help clinicians in taking decisions related to coagulation disorders. Several studies have stated that the time until analysis of samples for PT and APTT can be prolonged by appropriately storing samples at -20°C or -80°C, to give fairly accurate and reliable results.

These tests help clinicians determine if patients have a blood clotting disorder, bleeding disorder, or risk of excessive bleeding during surgery and to find out the efficacy of specific anticoagulant drugs. These tests are recommended to patients who are taking any anticoagulation therapy or have any bleeding disorder history. Sometimes, samples have to be stored before an analysis can begin. Several coagulation studies have reported the storage temperature and time for plasma samples. A study by Feng et al. demonstrated that samples for PT/INR can be stored in the refrigerator and at room temperature for 24 hours and those for APTT can be stored in the refrigerator for 12 hours and at room temperature for eight hours [6]. In another study, Zhao et al. showed that the storage time for PT/INR can be up to 24 hours in both room temperature and refrigerator and that for APTT is up to eight hours either at room temperature or refrigerator [8]. PT and APTT can be stored and tested for accurate results for eight hours at room temperature, and for PT, the upper limit of time reaches up to 24 hours [9]. Furthermore, Geelani et al. reported that PT samples could be stored for 24 hours at room temperature and in the refrigerator, but for APTT, the upper limit of a safe storage time is four hours [10].

Similarly, Oddoze et al. found that the storage time for APTT is six hours at room temperature or in the refrigerator [11]. Van Geest-Daalderop et al. stated that the time interval for PT/INR determination is six hours in the refrigerator, at room temperature [12]. All these studies prove that the duration for analyzing patient plasma can be extended, extracting accurate and reliable results. Thus, in this study, we investigated the stability of plasma samples after thawing storage for 24 hours at -20°C and -80°C in the freezer for PT, INR, and APTT tests and acceptable transition time up to which the samples can be analyzed for accurate and precise results. A total of 30 samples from healthy volunteers were analyzed for the baseline values at the time of collection and then were stored for 24 hours in -20°C and -80°C freezers. The samples were then tested after thawing for 15, 30, 60, and 120 minutes.

In our study, the difference between the mean values of the PT and INR of the samples stored at -20°C and -80°C for 24 hours and analyzed after thawing till 120 minutes and the baseline values was not statistically significant. Hence, the samples can be stored at either -20°C or -80°C and can be tested for PT and INR up to 120 minutes after thawing. The difference between the mean values of the APTT of the samples stored at -20°C for 24 hours and analyzed after thawing up to 30 minutes was not statistically significant, whereas the difference was significant for the samples tested after 30 minutes and up to 120 minutes. Similarly, the difference between the mean values of the APTT of the samples stored at -80°C for 24 hours and analyzed after thawing for up to 60 minutes was not statistically significant, but the difference was significant for the samples tested after 60 minutes and up to 120 minutes. This implies that the samples can be analyzed for APTT up to 30 minutes of thawing when stored at -20°C and up to 60 minutes when stored at -80°C.

Patient samples for coagulation may not be immediately tested due to various reasons, such as lack of laboratory facilities, financial constraints, and instrument-related issues, and hence there is a need to batch samples for processing. This study signifies the stability of the plasma samples in -20°C and -80°C freezers and the time up to which they can be tested reliably after thawing. Usually, PT/APTT are ordered in pairs using the same plasma specimen. Hence, if a clinician orders PT and APTT for a patient sample, based on the results obtained in the study, if the sample was stored at -20°C freezer, the sample must be tested within 30 minutes of thawing. Similarly, if a -80°C freezer was used, the sample must be tested within 60 minutes of thawing. As another option, the samples can be separately stored and tested for each test, but testing a single sample for both PT and APTT saves time and effort, and patient care decisions need not be delayed.

## Conclusions

Our findings show that plasma samples for PT and INR may be acceptable for assessment up to 120 minutes when stored at -20°C or -80°C for 24 hours. In the case of APTT, the plasma sample can be used for assessment up to 30 minutes after thawing when stored at -20°C. However, the stability of the plasma sample for APTT evaluation increases up to 60 min after thawing when stored at -80°C. PT and APTT tests are conducted primarily in pairs, using the same plasma specimen. Therefore, if the same sample was stored at -20°C for PT and APTT assessment, then the sample must be used within 30 minutes or within 60 minutes when stored at -80°C. If indicated and necessary, a separate aliquot must be made for each test and stored separately. This study could help laboratory professionals define a clear guideline for sample storage requirement after thawing for PT and APTT.
